# Previously reported and here added cases demonstrate euploid pregnancies followed by PGT-A as “mosaic” as well as “aneuploid” designated embryos

**DOI:** 10.1186/s12958-023-01077-7

**Published:** 2023-03-08

**Authors:** Norbert Gleicher, Pasquale Patrizio, Lyka Mochizuki, David H. Barad

**Affiliations:** 1 The Center for Human Reproduction, New York, NY USA; 2 The Foundation for Reproductive Medicine, New York, NY USA; 3 Stem Cell Biology and Molecular Embryology Laboratory, Rockefeller University, New York, NY USA; 4 Department of Obstetrics and Gynecology, Vienna University School of Medicine, 1009 Vienna, Austria; 5 Department of Obstetrics, Gynecology, and Reproductive Sciences, University of Miami, Miller School of Medicine, Miami, FL USA

**Keywords:** In vitro fertilization (IVF), Mosaicism, Aneuploidy, Preimplantation genetic testing for aneuploidy (PGT-A)

## Abstract

**Background:**

After the longest time opposing all transfers of embryos by preimplantation genetic testing for aneuploidy (PGT-A) diagnosed as “chromosomal-abnormal,” the field has over recent years slowly been moving toward selective transfers of by PGT-A as “mosaic” diagnosed embryos, but is still rejecting transfers of embryos by PGT-A defined as “aneuploid.”

**Methods:**

Upon review of the literature, we report published cases of euploid pregnancies following transfers of PGT-A as “aneuploid” diagnosed embryos and add several additional, ongoing cases at our center.

**Results:**

Among the published cases from our center, we identified seven euploid pregnancies from “aneuploid” embryos, four of which preceded the PGT-A industry’s 2016 switch from binary “euploid” – “aneuploid” reporting to “euploid,” “mosaic,” and “aneuploid” reporting. That those four cases post 2016 PGT-A definition involving “mosaic” embryos, therefore, cannot be ruled out. Since then, we recently established three additional ongoing pregnancies from transfers of “aneuploid” embryos which still await confirmation of euploidy after delivery. A recent fourth pregnancy from the transfer of a trisomy 9 embryo miscarried before a fetal heart. Outside our own center’s experience, the literature revealed only one additional such transfer, involving PGT-A as a “chaotic-aneuploid” diagnosed embryo with six abnormalities, leading to normal euploid delivery. In reviewing the literature, we furthermore demonstrate why current PGT-A reporting that differentiates between “mosaic” and “aneuploid” embryos based on relative percentages of euploid and aneuploid DNA in a single trophectoderm biopsy of on average 5-6 cells, is biologically non-sensical.

**Conclusion:**

Basic biological evidence and a clinically still very limited experience with transfers of PGT-A as “aneuploid” labeled embryos demonstrate beyond reasonable doubt that at least some “aneuploid” embryos can lead to healthy euploid births. Therefore, this observation establishes beyond reasonable doubt that the rejection of all “aneuploid” embryos from transfer reduces pregnancy and live birth chances for IVF patients. Whether (and to what possible degree) pregnancy and live birth chances differ between “mosaic” and “aneuploid” embryos, remains to be determined. The answer will likely depend on the aneuploidy(ies) of an embryo and to what degree percentages of “mosaicism” in a single, on average 5/6-cell trophectoderm biopsy can reflect the ploidy-status of a complete embryo.

## Introduction

Preimplantation genetic testing for aneuploidy (PGT-A) during in vitro fertilization (IVF) is becoming increasingly controversial [1–5]. After the births of hundreds of chromosomal-normal offspring following transfers of by PGT-A as chromosomal-abnormal defined embryos [6–8], even some of the most ferocious proponents of PGT-A have finally started to rescind their opposition to transfers of what current PGT-A practice defines as “mosaic” embryos [9, 10]. Indeed, even the PGDIS (Preimplantation Genetic Diagnosis International Society) came close to an endorsement of transferring “mosaic” embryos [11]. Claims that all by PGT-A as “aneuploid” diagnosed embryos can*not* lead to normal live births, however, have mostly prevented such transfers, even though, as we will here demonstrate in this review of the subject, biological facts regarding preimplantation-stage human embryos and the still limited clinical data involving such transfers clearly demonstrate that at least some by PGT-A as “aneuploid” diagnosed embryos can lead to normal pregnancies.

## What the literature says

Though proponents of PGT-A claim that a single trophectoderm biopsy (TEB) can distinguish between “mosaic” and “aneuploid” embryos, a single, on average 5-cell TEB, for biological as well as mathematical reasons, simply cannot discriminate between “mosaic” and “aneuploid” embryos [1, 4, 5]. A biological main reason is that TE and fetus derive from different cell lineages (extraembryonic and embryonic, respectively), which at preimplantation stages do not always correspond in respective ploidy. Both of these lineages downstream from the blastocyst stage, in addition, differ in their respective abilities to exclude aneuploid cells and, thereby, allow for selective self-correction in the embryonic lineage of the epiblast, while failing to do so in the extraembryonic lineage-producing trophectoderm and, ultimately, the placenta [12]. This difference is documented by the observation that chromosomally euploid and perfectly normal newborns still deliver with placentas with, often, considerable confined placental aneuploidy and an amalgam of genomic mutations [13]. Unsurprisingly, mathematical modeling, therefore, demonstrated indisputably that, even under the most favorable statistical assumptions, a 5-cell TEB (this is the number of average TE cells in a single biopsy claimed in the literature) cannot inform on the status of a complete embryo. To do so would require over 20 cells [14].

An even more important biological argument against differentiating between “mosaic” and “aneuploid” embryos when it comes to their transferability lies, however in how the PGT-A industry *incorrectly* defined “mosaicism” since 2016 [1, 4, 5]. While under uniform biological consensus, this term describes a single organism (in this case an embryo) that contains more than a single normal 46, XX, or 46, XY cell lineage, PGT-A laboratories describe an embryo *incorrectly* as “mosaic” (and, therefore the use of quotation marks) if only a single TEB of only approximately 5 cells contains more than a single normal 46, XX or 46, XY cell lineage.

The difference between the correct biological definition of mosaicism and the PGT-A definition, therefore, in itself, is disqualifying PGT-A from defining the correct ploidy status of an embryo after a single TEB at blastocyst-stage. It, indeed, does not take special genetics expertise to understand that, for this reason alone, a PGT-A diagnosis of “euploidy” or “aneuploidy” is practically worthless: “Euploidy,” even if all 5 biopsied cells are euploid, does not mean that “aneuploid” cells may not exist elsewhere in the embryo. The correct diagnosis of this embryo then, however, would be mosaicism, and not “euploidy.” Similarly, a diagnosis of “aneuploidy,” based on all 5 cells of a TEB being aneuploid, is worthless because most of the rest of the embryo may be euploid if only an island of aneuploidy was accidentally biopsied. Since a large majority of aneuploidies at blastocyst-stage are of mitotic origin, they are clonal and insular. Here, too, the correct diagnosis of the embryo would be mosaicism, and not “aneuploidy.” Considering how much more frequent mitotic than meiotic aneuploidies are, one is left with the conclusion that most PGT-A diagnoses of “euploidy” and “aneuploidy” are really mosaic embryos, a conclusion also supported by the reported prevalence of aneuploid cells in ca. 80% of embryos at blastocyst-stage [12].

Further indisputable conclusions, therefore, are that current PGT-A practice greatly exaggerates diagnoses of euploidy and aneuploidy and greatly underestimates diagnoses of mosaicism. A large majority of embryos undergoing PGT-A, including “euploid” as well as “aneuploid” embryos will, therefore, contain a mixture of euploid and aneuploid cells, with the ratio between cell lineages being variable. Then such variability, of course, raises the question of whether differences in percentages between euploid and aneuploid lineages matter.

Under the assumption that these differences do matter, some PGT-A laboratories have started to differentiate in their reports between “low” and “high” “mosaicism,” making the argument that embryos with lower percentages have better pregnancy and live birth rates [10, 15]. One of these studies was, however, refuted by a corrected reanalysis of that paper’s own data [16]. More importantly, however, as the previous discussion of the correct definition of mosaicism should already have exposed, considering ratios between euploid and aneuploid cell lineages in a 5-cell biopsy, simply, make no sense and cannot be predictive for the complete embryo. The only information a “mosaic” PGT-A result with absolute certainty provides is the assurance that this embryo really is mosaic. Whether within this relatively small group of mosaic embryos stronger presence of the aneuploid lineage means poorer outcome, is currently undetermined and cannot be completely precluded but, whatever that ratio may be, the correct biological definition of mosaicism establishes beyond reasonable doubt that the ratio found in a 5-cell TEB does *not* represent the likely ultimately important ratio for the complete embryo.

A purely empirical assessment of what currently is known, therefore, allows for the conclusion that any judgment of current PGT-A practice must conclude that restrictions of transferability of embryos based on current PGT-A definitions of “euploid,” “mosaic,” and “aneuploid,” have no biological, mathematical, or ethical basis and, therefore, should be withdrawn. Our center, therefore, as of this point in time recommends on chromosomal grounds only the withholding from the transfer of embryos with reported aneuploidies known to survive and, even this restriction, may turn out to be excessive.

## Confirmed cases of healthy births following transfers of “aneuploid” embryos

### Our center’s registry data

As noted in the abstract, we reported four normal pregnancies/births following what then were considered “aneuploid” embryos in 2015 [16]. Since our center as of this point started accepting selective chromosomal-abnormal embryos from other IVF centers for transfer, our center in 2016 initiated a registry for all transfer cycles with chromosomal-abnormal embryos performed at the center. Table 1 summarizes three patients who in their respective IVF cycles, produced only chromosomal-abnormal embryos defined as “aneuploid.” Those embryos at the IVF centers where they had been produced were not considered transferrable.

**Table 1:** Characteristics of 2 “aneuploid” embryo transfers leading to normal pregnancy/delivery*

Patient	TEB result	Diagnostic platform	Transfer date	IVF cycle outcome	Chromosomal outcome
1	48, XX, + 14, −18	Microarray	6/10/2020	NSVD, 2/26/2021	46, XX
2	46, XY, del/dup 20; 45, XY, −21	Microarray	6/28/2021	NSVD, 3/14/2022	46, XY

*A 3rd pregnancy, transferred on 2/10/2020 with a single “aneuploid” embryo (47, XY, + 16), experienced a potentially preventable pregnancy loss shortly following an amniocentesis that demonstrated a normal 46, XY karyotype by microarray [5]

*NSVD* normal spontaneous vaginal delivery, *EDC* expected date of confinement

Our center offered transfers of selected abnormal embryos since 2014, reporting the four first normal pregnancies in the world following such transfers in 2015 [16]. This report preceded the 2016 decision of the PGT-A community to switch the diagnosis of aneuploidy in blastocyst-stage embryos to next-generation sequencing and, with it, move from binary “euploid”-” aneuploid” to trinary “euploid,” “mosaic,” “aneuploid” outcome reporting. Reported by the PGT-A laboratory as “aneuploid, we, therefore, cannot rule out that, after 2016, these embryos might have been labeled as “mosaic.”

While initially there was unanimity that “euploid” was defined by < 20% “aneuploid” lineage DNA, “mosaic” by 20-80%, and “aneuploid” by > 80%, this consensus has since dissipated. Percentages used in different laboratories currently, indeed, differ to significant degrees, creating a confusing picture, resulting in embryos having different potential diagnoses at different PGT-A laboratories. Due to all of this confusion, increasing numbers of laboratories have, therefore, indeed returned to binary “euploid”- “aneuploid” reporting, with the cut-off between the two placed at either 40% or 50% “aneuploid” lineage DNA.

We recently updated our center’s ongoing registry of “chromosomal-abnormal” embryos transferred since 2015 in two reports [7, 8, 12]. Based on the above-summarized reasoning, our center has never differentiated between “mosaic” or “aneuploid” PGT-A diagnoses. Moreover, as we recently explained elsewhere [8], we try to avoid all three designations of embryos and prefer the use of the term “chromosomal-abnormal,” with the understanding that this is only a temporary designation of embryos with very limited clinical significance.

So far, we in-toto established 19 clinical pregnancies in women after exclusively transferring only “aneuploid” embryos including the four deliveries before 2016. Among those, three produced chromosomal-normal pregnancies, and two delivered and were reported in earlier accountings of our center’s patient registry [7, 12]. A third patient after the transfer of an “aneuploid” embryo demonstrated at amniocentesis a normal 46, XY pregnancy, but miscarried as a likely complication of the procedure shortly following the procedure (Table 1). Eight pregnancies spontaneously miscarried, with the chromosomal abnormality in a transferred embryo corresponding to the chromosomal abnormality in miscarried products of conception, though two patients refused analysis of products of conception [7, 12].

Since the last report based on the registry [7], we established an additional four ongoing pregnancies from exclusively “aneuploid” embryos, three among those are still ongoing the most advanced pregnancy at the time of this report at 20 weeks gestation with a singleton pregnancy. Two other singleton pregnancies are at 13 and 8 weeks, respectively. One pregnancy following the transfer of a trisomy 9 was unfortunately miscarried.

### Outside data

Colleagues from the Department of Obstetrics and Gynecology at the University of Rochester Medical Center recently reported a single live birth following the transfer of a “chaotic-aneuploid” embryo reported by PGT-A to demonstrate 6 chromosomal abnormalities [17]. They in their paper also reported that the PGT-A laboratory where the test was performed (IGENOMIX, Florida, USA) recently issues a circular to IVF centers in which it announced that re-biopsy of “chaotic-aneuploid” embryos (6 or more abnormalities) in their laboratory had revealed an approximately 40% “normal-euploid” rate, very much reaffirming the highly questionable clinical utility of PGT-A in association with routine IVF practice. Our center reported significant discrepancies between biopsies in the same embryos already in 2015 [16].

As IVF centers have increasingly started transferring “mosaic” embryos since 2015, a majority of IVF centers, still, do not transfer embryos unless signed out as “euploid.” The above-noted case out of Rochester, moreover, is the first we have become aware of, in which another IVF center other than ours transferred an “aneuploid” embryo. That not more successful cases of transfers of “aneuploid” embryos have been reported, therefore, should not surprise. A commentary in association with the Rochester case well summarized the current status quo [18].

## Discussion

As currently practiced, PGT-A is based on an unconventional 2016 guidance by a small society in an unreferenced and unsigned e-mail to membership (recently removed from the society’s website) [1]; yet, inexplicitly, this guidance to this day dictates the practice of PGT-A and, with it, the practice of IVF. It is estimated that approximately half of all U.S. IVF cycles are currently accompanied by PGT-A. As already noted, this PGDIS guidance introduced an incorrect definition of “mosaicism” to the procedure which, to this day, is driving the current confusion in the reporting of PGT-A results and, therefore, ultimately in the decision-making process as to which chromosomal abnormal embryos after PGT-A can or should not be transferred.

Most PGT-A laboratories still report embryos as “aneuploid” (and, therefore, as “untransferable”) if a single TEB demonstrates between 80 and 100% aneuploid DNA. If the aneuploidy is mitotic, even 100% aneuploid TEBs, however, more likely reflect a mosaic than a truly aneuploid embryo. Only meiotic aneuploidies, representing a relatively small minority of aneuploidies of embryos at preimplantation stages, can be expected to be present in every cell of an embryo. Only meiotic aneuploidies, therefore, can be reliably diagnosed with a 5-cell TEB.

Following the publication of increasing evidence, even proponents of PGT-A now no longer can reject that “mosaic” embryos (under the incorrect PGT-A definition) produce identical live birth rates to PGT-A “normal” or even untested embryos [9–11]. These same authors for many years and until very recently have, however, vehemently argued against the transfer of *any* chromosomal-abnormal embryo. Their authority in calling for “mosaic,” but not “aneuploid” embryos (under PGT-A criteria), to be transferred, therefore, must be viewed as limited. By continuing to argue against transfers of “aneuploid” embryos, they, indeed, just continue the waste of valuable human embryos, a consequence of PGT-A they have been responsible for over many years.

That PGT-A laboratories now utilize different percentage cut-offs of aneuploid DNA to reach formal diagnostic designations for embryos confirms the limitations of PGT-A. One, therefore, must conclude once again that such measurements in a 5-cell TEB are, simply, not precise enough in determining whether an embryo should be transferred or not [11]. Not only is there no logic behind this kind of reporting, but it even further confuses the interpretation of PGT-A results because what in one laboratory may now be reported as a “normal” embryo, in another may be reported as either a “low- “or “high-mosaic,” with many PGT-A laboratories recommending that only “low-mosaic” embryos be transferred.

PGT-A reporting, thus, has become an uninterpretable conundrum for most IVF centers, of course, further aggravated by the fact that embryos have the ability to self-correct downstream from blastocyst stage. It was a mouse study that, first, demonstrated that self-correction in embryos (derived from the embryonic cell lineage) inversely correlated with the percentage of aneuploidy in the epiblast [19]. A few years later, self-correction was also demonstrated in human embryos [12].

As “high-mosaic” and “aneuploid” embryos are, still, widely refused transfer, PGT-A in such cases continues to deprive patients of significant pregnancy and live birth chances and often contributes to their premature referral into third-party egg donation [1]. Since the argument that by PGT-A criteria “aneuploid” embryos cannot result in chromosomal-normal births is often used in defense of refusing embryo transfers, we considered it important to present here reported update on the subject.

## Data Availability

All data generated or analyzed during this study are included in this published article. The datasets used and/or analyzed during the current study are available from the corresponding author upon reasonable request.

## Abbreviations

PGT-A: Preimplantation genetic testing for aneuploidy
IVF: In vitro fertilization
TEB: Trophectoderm biopsy
PGDIS: Preimplantation Genetic Diagnosis International Society
